# Recent Advances in Biomarkers and Potential Targeted Therapies in Head and Neck Squamous Cell Carcinoma

**DOI:** 10.5402/2012/715743

**Published:** 2012-02-15

**Authors:** Eric J. Yavrouian, Uttam K. Sinha

**Affiliations:** Department of Otolaryngology, Keck School of Medicine of USC, University of Southern California, Los Angeles, CA 90089, USA

## Abstract

Head and neck squamous cell carcinoma (HNSCC) is a devastating tumor of the upper aerodigestive tract with no significant change in treatment modality or improvement in survival over the last several decades. Biomarkers are important biological molecules that can be utilized in tumor detection, prognosis, and as targeted therapies. There are several important biomarkers and potential targets in the forefront, including biomarkers of tumorigenesis, signal transduction molecules, proteins involved in angiogenesis, and oncogenic viruses. The clinical applications of these biomarkers are in various states from *in vitro* and *in vivo* models, phase II and III clinical trials, to accepted modes of treatment in patients with HNSCC. Given the potential improvement in prognosis that biomarkers and their targeted therapies may have on the treatment of HNSCC, their investigation is both important and essential.

## 1. Introduction

Squamous cell carcinoma is the most common cancer arising in the head and neck with devastating effects on communication, swallowing, and, most importantly, survival. Head and neck squamous cell carcinoma (HNSCC) is an epithelial cancer arising in the upper aerodigestive tract for which new biomarkers and targeted therapies are needed for rapid diagnosis and treatment. It is a tumor occurring most commonly in the oral cavity, oropharynx, hypopharynx, and larynx [1]. In 2000, there were 480,000 new cases of HNSCC each year worldwide and HNSCC compromised approximately 4% of malignant neoplasms [2, 3]. In the United States, approximately 36,000 cases are expected to occur in 2010 with an estimated 8,000 deaths [4]. Men are at greater risk than women with the two greatest risk factors consistently being tobacco and alcohol use. Traditionally, the major treatment for HNSCC has been surgical resection with postoperative chemoradiation. This treatment modality has not significantly changed over the past 30 years with only minimal improvement in survival. Overall survival ranges from 70 to 85% for patients presenting with early-stage disease (stage I and II) to 30–40% for advanced-stage disease (stages III and IV) [5]. Thus, new biomarkers for earlier detection as well as targeted therapies are essential.

 Tumor biomarkers are a novel avenue with which one can improve early detection of tumor, improve monitoring and treatment, and ultimately increase disease survival. Biomarkers are biological molecules that when measured can correlate with the presence or absence of primary disease and metastasis, predict disease prognosis, or offer a potential target for specified therapies. A diverse range of biomarker types exist and can be measured including changes in the host genome, differential expression of proteins involved in processes such as angiogenesis, and the presence of viral infections. In recent years, several new biomarkers have been identified and are currently being studied for their effectiveness in HNSCC detection, prognosis, and treatment.

## 2. Tumor Pathogenesis

 Neoplasia in the head and neck is a multistep process with sequential mutations in genes responsible for tumor surveillance. A microsatellite analysis of allelic alterations demonstrated that with the accumulation of genetic mutations, one can follow the transformation from simple squamous hyperplasia to severe dysplasia, and, ultimately, invasive squamous cell carcinoma [6]. p53, a tumor suppressor gene, has been implicated in the early pathogenesis of HNSCC, as it controls cell growth through regulation of the cell-cycle and apoptosis [7–9]. In a study analyzing HNSCC patients with a history of tobacco and alcohol use, Brennan et al. found a significantly higher proportion of patients with mutations of p53 and other distinct sites when compared to nonsmokers and nondrinkers [10]. p53 mutations have been found in up to 50% of HNSCC patients and have been shown to be associated with decreased survival [8].

In addition to p53, mutations in the retinoblastoma (Rb) gene are involved in the pathogenesis of HNSCC. p16^INK4A^, a major target of the Rb pathway, is a tumor suppressor gene; its function is inhibited through a variety of pathways including loss of heterozygosity (LOH) of chromosome 9p21 where it is located. LOH of 9p21 is seen in 30% of premalignant lesions and up to 80% of malignant lesions [11]. LOH in other locations, including LOH of chromosome 3p, has also been associated with tumorigenesis. Lee et al. examined mutations in eight different HNSCC cell lines and found that three candidate oncogenes encoded on chromosome 3p (ALS2CL, EPHA3, and CMYA1) were mutated, implying that LOH of chromosome 3p is also associated with HNSCC [12]. An ongoing clinical trial of the tyrosine kinase inhibitor Erlotinib and oral cancer is assessing the clinical outcomes in patients with LOH of 9p and 3p compared to patients with normal DNA [13]. LOH of both 3p and 9p has also been shown to help differentiate dysplastic and hyperplastic lesions that are likely to progress to carcinoma [11, 14, 15]. Despite the fact that p53 and Rb mutations as well as LOH at 3p and 9p are clinically relevant biomarkers of HNSCC, they have yet to be applied in daily practice.

In addition to the p53 and Rb genes, sphingosine kinases (SphKs) have recently emerged as molecules of interest in HNSCC. By regulating levels of ceramide, sphingosine, and sphingosine-1-phosphate (S1P), SphK influences cells to enter proliferative states as opposed to apoptotic states [16–19]. SphK1, the SphK isozyme most studied in neoplastic diseases, has been shown to be upregulated in HNSCC with overexpression in advanced stage and recurrent tumors. Use of small molecular inhibitors or siRNA's targeting SphK1 has been demonstrated to sensitize cells both in *in vitro* and *in vivo* studies to radiation [20, 21]. As a cell cycle regulator overexpressed in HNSCC patients, SphK1 provides another prospective avenue in the treatment of HNSCC.

## 3. Signal Transduction Biomarkers

 One of the most well-known biomarkers in HNSCC is the Epidermal Growth Factor Receptor (EGFR). EFGR is a receptor tyrosine kinase involved in multiple downstream signaling pathways influencing cell growth, angiogenesis, and invasion [22]. Downstream EGFR signaling activates the mitogen-activated protein kinase (MAPK) pathway as well as the phosphatidylinositol 3-kinase (PI3-K)/protein kinase B (Akt) pathway [23]. Activation of the MAPK pathway leads to increased expression of antiapoptotic proteins like Bcl-x_2_ and inhibition of proapoptotic proteins like BAD [24]. Signaling through the PI3-K/Akt pathway ultimately leads to inhibition of the tumor suppressor gene p53 [25]. All of these result in a proliferative state and inhibition of tumor suppressor function.

Overexpression of EGFR in HNSCC has been associated with poorer overall survival and recurrence, and up to 90% of HNSCC patients express EGFR [22, 26–28]. With EGFR overexpression implicating a poor prognosis, it was one of the first biomarkers targeted as a potential therapy for HNSCC. Cetuximab, a monoclonal antibody directed against the extracellular receptor domain of EGFR, blocks ligand binding and subsequent downstream signaling, in addition to its role in the long-term downregulation of the receptor expression [29–33]. It has been the most successful targeted therapy applied in HNSCC to date. In a phase III clinical trial by Bonner et al., cetuximab in combination with radiotherapy provided an overall survival benefit of an additional 20 months compared to radiation alone [34, 35]. There have also been several clinical trials comparing chemotherapy alone or in combination with cetuximab. In the phase III trial, Erbitux in first-line treatment of recurrent or metastatic head and neck cancer (EXTREME), 442 patients with recurrent or metastatic HNSCC were randomized to receive either platinum/5-FU alone or cetuximab plus platinum/5-FU. Results showed an increase in response rate from 20% in the chemotherapy group to 36% in the chemotherapy plus cetuximab group, with an overall survival increase from 7.4 months in the chemotherapy group to 10.1 months in the chemotherapy and cetuximab group [36]. Another phase III clinical trial from the Radiation Therapy Oncology Group (RTOG) comparing concurrent chemoradiation and Cisplatin versus concurrent chemoradiation with Cisplatin and cetuximab in patients with stage III and IV HNSCC is pending [13].

 In addition to monoclonal antibodies targeting EGFR, small molecule tyrosine kinase inhibitors (TKIs) are also capable of inhibiting EGFR function. Phase I and II clinical trials in patients with recurrent or metastatic HNSCC have been conducted on the TKIs erlotinib and gefitinib. With erlotinib, these trials show an overall survival range of six to eight months and a response rate from 4% to 21%. With gefitinib, phase I and II clinical trials have demonstrated an overall survival ranging from six to eight months, with a response rate of 1% to 15% [37–41]. While studies of erlotinib and gefitinib have demonstrated some response in HNSCC, results of phase III clinical trials on TKIs are still pending [13].

## 4. Biomarkers of Angiogenesis

 In addition to their potential as useful biomarkers in HNSCC, markers of angiogenesis provide a therapeutic opportunity in HNSCC. Angiogenesis play an important role in tumor growth and progression [42]. Without new vessel growth, tumors are unlikely to grow beyond 3 mm [43–46]. There are three families of receptor tyrosine kinases involved in angiogenesis: the ephrins and the Eph receptors, the angiopoetin family, and the vascular endothelial growth factor (VEGF) [47]. Of these three receptor tyrosine kinase families, VEGF is the most extensively studied. VEGF has been shown to be overexpressed in tumor cells compared to normal cells; this overexpression is associated with a 1.88-fold increased risk of death and is also associated with lymph node metastasis [48–50].

There are several antiangiogenic targets currently undergoing clinical trial. Tyrosine kinase inhibitors of the VEGF receptors halt their intracellular signaling. Several of these small molecule tyrosine kinase inhibitors, sunitinib, sorafenib, vandetanib, semaxanib, and foretinib, are undergoing phase II clinical trials [13]. Sunitinib was studied by Machiels et al. in a phase II clinical trial of 38 HNSCC patients in which it was given as a palliative treatment; they achieved a disease control rate of 50%. However, due to several complications that occurred including bleeding, skin ulceration, and fistulas, they recommended further study of the drug to assess which patients would benefit [51]. Sorafenib's effect was studied in recurrent/metastatic HNSCC and nasopharyngeal carcinoma with a response rate of 3.7%. Given that it is a multikinase inhibitor, its effect cannot be attributed only to its antiangiogenic activity [52]. Semaxanib was also studied in HNSCC as a single agent, but its use was discontinued due to several adverse affects and its difficulty with administration [53]. Bevacizumab, a monoclonal antibody against the VEGFA ligand, has been reviewed in phase I and II clinical trials with convincing evidence of antitumor activity in HNSCC patients when combined with erlotinib [54, 55]. Vandetanib, a VEGF receptor inhibitor, is one of the drugs currently undergoing clinical trial. This drug is unique in that it acts as an inhibitor of the VEGF receptor, the EGFR receptor, and the rearranged during transfection (RET) tyrosine kinases. *In vitro*, it has an inhibitory effect on HNSCC cells; however, results of phase II clinical trials are currently under investigation [13, 56].

Another receptor tyrosine kinase of potential interest in HNSCC is the Eph receptor family and its ligands, the Ephrins. This group of proteins has an important role in many physiologic processes including cell aggregation and migration, angiogenesis, and vascular network development [57–59]. EphB4 and its sole ligand EphrinB2 are overexpressed in all primary and metastatic tumors, with EphB4 overexpression correlating with advanced stage disease and lymph node metastasis. *In vivo*, EphB4 has also been demonstrated to provide a survival advantage to tumor cells, and, its inhibition has been shown to decrease the survival of the HNSCC tumor cells. Furthermore, an analysis of HNSCC patients and EphB4/EphrinB2 expression demonstrated that overexpression of EphB4 and EphrinB2 was associated with a significantly worse overall survival [60–63]. Given that EphB4 and EphrinB2 are overexpressed in HNSCC and that this is associated with worse overall survival, EphB4 and EphrinB2 are potentially useful biomarkers and may provide another target for HNSCC treatment.

## 5. Oncogenic Viruses

 In recent years, the human papilloma virus (HPV) and its link with HNSCC, particularly in oropharyngeal tumors, has been illustrated. Not only can HPV be used as a biomarker of prognostic significance, but also as a preventative target. Of the several types of HPV, type 16 is most commonly associated with HNSCC [64–66]. HPV is a double-stranded DNA virus that encodes several proteins, among which are three oncoproteins: E5, E6, and E7 [67]. The carcinogenic effect of HPV is mainly due to oncoproteins E6 and E7. HPV E6 expression ultimately leads to disruption in function of p53 and its antitumor protective effect [68]. Similarly, oncoprotein E7 inhibits the Rb protein and its tumor suppressor function [69].

 Using either PCR or *in situ *hybridization assays, HPV status can be detected in head and neck tumors [65, 70]. The RTOG 0129 clinical trial showed that 64% of oropharyngeal tumors were HPV-positive and that this subset of patients was younger, less likely to use tobacco, and had smaller tumors [71]. Thus, HPV-positive HNSCC patients have a significantly improved survival and treatment outcome independent of the mode of treatment [72–74]. In a prospective study evaluating HNSCC patients treated with induction chemotherapy and radiation, patients with HPV-positive tumors had a higher response after treatment, 84% in the HPV-positive group versus 57% in the HPV negative tumors. Two-year overall survival in patients with HPV-positive tumors was 95% compared to 62% in patients who were HPV negative [72].

HPV status can be used as a biomarker of improved prognosis; however, it also has a potential application in HNSCC management, both with preventative vaccination and as a targeted therapy. In 2006, the HPV vaccine, Gardasil was approved by the US Food and Drug Administration and recommended by the Center for Disease Control (CDC) in girls and young women to prevent cervical cancer. The vaccine targets HPV types 6, 11, 16, and 18. Since then, in 2009, the CDC has also recommended the vaccine in young men aged nine through 26 for the prevention of genital warts and HPV-associated cancers including HNSCC [75]. In an effort to utilize HPV status as a mode of treatment, Wu et al. have developed an HPV vaccine that enhances the T-cell immune response in mice with HPV-positive tumors [76]. This vaccine has potential as a therapeutic modality in patients with HPV-positive tumors to further improve survival.

## 6. Biomarkers in Saliva

 With advances in the search for biomarkers in HNSCC, many researchers are interested in finding potential biomarkers in one of the most easily accessible tissues, patients' saliva—mainly through the study of differential gene expression, expression of proteins such as telomerase, and through mass spectrometry. In a study reviewing 82 candidate genes, Sethi et al. were able to demonstrate the differential expression of genes present in the saliva of HNSCC patients as compared to controls. The expression of the genes, PMAIP1 and PTPN1, correlated with HNSCC in 27 patients compared to 10 control patients [77]. Telomerase, a protein involved in emergence from cellular senescence, has an increased degree of activity in malignant cells. With PCR-based techniques using oral rinses from HNSCC patients, Califano et al. was able to show increased telomerase activity in the saliva of HNSCC patients compared to controls [78]. In recent years, with advances in the field of proteomics and mass spectrometry, one is able to simultaneously analyze multiple proteins found in oral rinses in an attempt to discover potential tumor biomarkers [79]. There is a significant interest in developing a study model with which HNSCC can be detected in saliva, however, one biomarker or method of detection has yet to be applied systemically outside of the laboratory setting.

## 7. Discussion

 With further advances in our understanding of HNSCC and its pathogenesis, HNSCC is a seemingly heterogeneous group of tumors rather than a single type with one appropriate treatment. With the emergence of several new biomarkers, one can improve detection of tumor, obtain prognostic information and offer new treatments in head and neck squamous cell carcinoma. As we delineate which biomarkers are overexpressed in patients, we can offer individual patients with HNSCC-specific prognostic information, and tailor treatments to patients based on the molecular profile of their tumor.

To date, few of these biomarkers are applied clinically for prognostic information. Although in many studies the overexpression of these biomarkers has been associated with poorer prognosis, they have yet to be applied clinically. There are no standardized techniques or clinical values developed to assess the expression of these biomarkers in HNSCC tumor samples. HPV status is unique among this group as it is now routinely tested in many patients with oropharyngeal tumors and carries with it an improved prognosis. Methods such as protein and DNA microarray technology can be used to assess the molecular profile of a tumor and test for multiple biomarkers simultaneously [80]. Thus far, microarrays have not been applied outside the laboratory setting in HNSCC.

EGFR expression, and its inhibition with antibodies such as cetuximab, presents an example of how biomarkers can be successfully used as indicators of prognosis and as therapeutic targets to improve survival. VEGF is likely the next biomarker to be targeted in HNSCC therapy in daily clinical practice. The tumor suppressor genes p53 and Rb, and regulatory proteins like SphK1 and EphB4/EphrinB2 offer both prognostic value and a means with which to detect malignant cells. However, the presence of these biomarkers is not routinely tested for in the clinical laboratory and therapies targeting these biomarkers have yet to be developed.

While there has been a great deal of advancement in biomarker detection and targeted therapies in HNSCC, there are still very important aspects of HNSCC we do not understand. In patients with HNSCC, there is an approximate 4% annual risk of developing a second primary tumor [81]. These second primary tumors are thought to be a result of “field cancerization” [82, 83]. A future area of interest would be to identify a biomarker of field cancerization and develop a target that would prevent recurrence or a second primary tumor.

HNSCC is a tumor that carries with it a significant morbidity and a very poor prognosis, especially in advanced disease. Therefore, the development of biomarkers that can play a role in the earlier detection of tumor cells, offer prognostic information and can be used as targeted therapies is crucial.
